# The clinical value of nanocarbon suspension and methylene blue in sentinel lymphatic tracing and pathologic fine sorting of penile cancer: a multicentre prospective study

**DOI:** 10.1080/07853890.2025.2603625

**Published:** 2025-12-19

**Authors:** Chengyi Liu, Tao Tao, Mingshan Yang, Fajun Pei, Mo Yan, Lingfeng Sun, Dongliang Li, Jun Han, Qixia Liu, Huimin Wan, Wei Liu, Shuangjie Li, Tonghui Weng, Xuexiang Li, Bo Pei, Yichen Wang, Xiaodong Bai, Guohui Wu, Changlin Sang, Nan Ye, Yang Su, Pengcheng Xu, Guangyuan Li

**Affiliations:** ^a^ Department of Urology, Lu’an People’s Hospital of Anhui Province, Lu’an Hospital of Anhui Medical University, Lu’an, China; ^b^ Department of Urology, The First Affiliated Hospital of USTC, Division of Life Sciences and Medicine, University of Science and Technology of China, Hefei, China; ^c^ Department of Urology, Anhui Provincial Cancer Hospital, Hefei, China; ^d^ Department of Urology, Shandong Cancer Hospital, Ji’nan, China; ^e^ Affiliated Tumor Hospital of Shandong First Medical University, Ji’nan, China; ^f^ Department of general surgery, Lu’an People’s Hospital of Anhui Province, Lu’an Hospital of Anhui Medical University, Lu’an, China; ^g^ Department of Urology, Shanghe People’s Hospital of Ji’nan, Ji’nan, China; ^h^ Department of Pharmacy, Lu’an People’s Hospital of Anhui Province, Lu’an Hospital of Anhui Medical University, Lu’an, China; ^i^ Anhui Medical University, Hefei, China; ^j^ Department of Urology, The Fourth People’s Hospital of Lu ‘an, Lu’an, China; ^k^ Shucheng People’s Hospital of Lu ‘an, Lu’an, China; ^l^ Huoshan County Hospital of traditional Chinese medicine, Lu’an, China; ^m^ Department of Urology, The First Affiliated Hospital of Anhui Medical University, Hefei, China; ^n^ Anhui Public Health Clinical Center, Hefei, China

**Keywords:** Nanocarbon suspension, methylene blue, lymphatic tracing, fine sorting, penile cancer

## Abstract

**Objective:**

To compare nanocarbon suspension and methylene blue as lymphatic tracers in inguinal lymph node mapping and sorting during surgery for penile cancer.

**Methods:**

This multicentre study included 62 patients (2020–2024); 33 underwent sentinel lymph node biopsy (SLNB) and 29 underwent inguinal lymph node dissection (ILND). SLNB patients were divided into control (no tracer), nanocarbon, or methylene blue groups (ChiCTR2200063416). Outcomes included lymph node detection rate (LNDR), number of lymph nodes per side (NOLNPIS), metastasis positive rate (PROLM), and operation time (OT). ILND patients were grouped into nanocarbon-assisted sorting (NLFLNSG) or traditional sorting (TLNSG), with comparisons of NOLNPIS, PROLM, OT, and lymphatic leakage rate (PLLR).

**Results:**

Both tracers effectively stained lymph nodes without adverse effects. During SLNB, methylene blue significantly improved LNDR (*p* = 0.013), NOLNPIS (*p* = 0.015), and reduced OT (*p* < 0.0001) compared to control, but PROLM showed no difference. In ILND, NLFLNSG yielded more lymph nodes than TLNSG (*p* = 0.043), with no significant differences in PROLM, OT, or PLLR.

**Conclusion:**

Nanocarbon and methylene blue are safe and effective for lymphatic tracing in penile cancer SLNB, improving node identification and surgical efficiency. Nanocarbon-assisted sorting in ILND increases lymph node retrieval, aiding pathological staging and treatment guidance.

## Introduction

Penile cancer is a rare malignancy of the urogenital system, accounting for less than 1% of adult cancers in developed countries. However, its incidence is relatively higher in regions such as India, South America, and Africa, where neonatal circumcision rates are lower [1,2]. The most common histological type is squamous cell carcinoma (SCC). Despite treatment, up to 25% of patients experience disease recurrence within the first year [3]. Lymphatic spread is the primary mode of metastasis, and lymph node involvement is a critical prognostic factor for both recurrence-free survival and overall survival [4]. Studies show that patients without regional lymph node metastasis have a 5-year survival rate of 95%–100%. However, when a single inguinal lymph node metastasis occurs, the survival rate drops to 80%. If multiple inguinal lymph nodes are affected, the survival rate further decreases to 50%. Once pelvic or para-aortic lymph node metastasis develops, the 5-year survival rate falls to 0% [5].

In clinical practice, when inguinal lymph node metastasis is suspected, sentinel lymph node biopsy (SLNB) or prophylactic inguinal lymph node dissection (ILND) is typically recommended. However, ILND is a highly invasive procedure with complication rates ranging from 10% to 78%, including lymphorrhoea, wound infection, and lower limb lymphedema [6,7]. As a result, SLNB has become widely used for diagnosing inguinal lymph node metastasis in penile cancer.

Lymphatic metastasis in penile cancer has two key characteristics. First, it progresses stepwise: tumor cells spread to superficial inguinal lymph nodes (unilateral or bilateral) before moving to deep inguinal lymph nodes, pelvic lymph nodes, and potentially retroperitoneal nodes or organs. Skip metastasis is rare. Second, the location of sentinel lymph nodes varies from individual to individual and is not fixed to a specific anatomic area, leading to a false-negative rate of approximately 25% [8]. To address these issues, dynamic sentinel lymph node biopsy has been introduced. By injecting a tracer around the tumor, sentinel lymph nodes can be stained and located, helping surgeons identify and remove them more accurately. Commonly used tracers include indocyanine green and 99Tcm-labeled colloid, which can be used alone or in combination [9–11]. However, these tracers carry radioactive risks and require expensive imaging and radiation detection equipment, increasing procedural complexity, surgery time, and the financial burden on hospitals and patients. Given the high incidence of penile cancer in countries and regions with limited healthcare resources, such devices are often not readily available. Therefore, developing new, efficient, and low-cost tracers is of significant clinical importance.

Recent studies have shown that nanocarbon suspension is a promising lymphatic tracer in cancers such as gastric, breast, endometrial, colorectal, and thyroid cancers [12–16]. Our preliminary research also indicated that local injection of nanocarbon suspension around the tumour in penile cancer effectively stains inguinal sentinel lymph nodes. This method helps distinguish sentinel lymph nodes from adipose tissue that is similar in shape, texture and colour, significantly enhancing surgical efficiency. The naked-eye effect of nanocarbon suspension eliminates the need for expensive equipment and carries no radioactive risks [17].

Building on our previous research, we further explored methylene blue as a more affordable tracer with similar visual properties, comparing it to nanocarbon suspension in sentinel lymph node biopsy for penile cancer. Sentinel lymph nodes are typically located in the superficial inguinal group, but ILND for penile cancer includes both superficial and deep lymph nodes. Therefore, it is important to assess whether these tracers can label deeper lymphatic pathways. Additionally, by combining lymphatic marking of tracers with fine lymph node sorting of postoperative specimens, we aim to evaluate its clinical value in ILND and provide more precise pathological staging for postoperative management.

### Clinical data and methods

For clinical trials, the protocol was approved by Chinese Clinical Trials Registry (Registration number: ChiCTR2200063416, website: https://www.chictr.org.cn/showproj.html?proj=178138)

#### Data sources and patient selection

This study included 62 patients diagnosed with penile cancer across 8 medical centers, from January 2020 to December 2024. Of these, 33 patients underwent SLNB, while 29 patients underwent ILND.

##### Inguinal sentinel lymph node biopsy group

Inclusion criteria:

- Patients diagnosed with penile cancer;
- Pathological staging ≥ pT1G2 and clinical staging ≥ cN0;
- Preoperative chest, abdominal, and pelvic CT scans revealed no distant metastasis or enlarged pelvic lymph nodes, irrespective of whether enlarged inguinal lymph nodes were palpated. However, the lymph nodes are mobile, smooth on the surface, and the maximum diameter is no more than 1.5 cm. Additionally, under the observation of color Doppler ultrasound of the groin, although the lymph nodes have increased in size, there are no abnormalities in their shape, internal echoes, or blood flow signals.

Exclusion criteria:

- Patients with severe cardiopulmonary dysfunction or other contraindications for surgery;
- Patients who, during the study, could not provide valid data due to relevant factors.

All patients underwent unilateral or bilateral inguinal sentinel lymph node biopsy. Prior to the procedure, patients were randomly assigned to the carbon nanoparticle group, methylene blue group, or control group without tracer injection based on whether carbon nanoparticles or methylene blue were injected.

##### Inguinal lymph node dissection group

Inclusion criteria:

- Patients diagnosed with penile cancer;
- Positive pathological results from inguinal sentinel lymph node biopsy;
- Positive pathological results from color doppler ultrasound-guided lymph node puncture;
- Diagnosed with N1∼N2 stage and still palpable enlarged lymph nodes after antibiotic treatment.

Exclusion criteria:

- Patients with severe cardiopulmonary dysfunction or other contraindications for surgery;
- Patients who, during the study, could not provide valid data due to relevant factors.

All patients underwent unilateral or bilateral ILND. Based on whether carbon nanoparticles were injected preoperatively and the method of post-surgical lymph node specimen collection, patients were randomly assigned to either the traditional lymph node sorting group(TLNSG) or the nanocarbon labelled fine lymph node sorting group(NLFLNSG).

All patients underwent partial penectomy or total penectomy with urethrocutaneous fistula formation. The study protocol adhered to the Declaration of Helsinki and was approved by the Medical Ethics Committee of the Affiliated Lu’an Hospital of Anhui Medical University (Approval No. 2020LL014). All patients provided written informed consent.

### Materials and methods

#### Preparation of nanocarbon suspension

Nanocarbon was prepared by ball milling activated carbon into ultrafine particles. The particles were sieved through a 100-mesh filter and then dried at 120 °C for 2 h. After sterilization under 2 atm pressures for 10 min, the final product consisted of smooth carbon particles with an average size of approximately 20 nm. To create the black suspension, polyvinylpyrrolidone (PVP) and physiological saline were added as stabilizers.

#### Injection method of nanocarbon suspension

After achieving satisfactory anaesthesia, 0.5 mL of either nanocarbon suspension (Chongqing Lummy Pharmaceutical Co., Ltd., Chongqing, China) or methylene blue (Jichuan Pharmaceutical Group Co., Ltd., Taixing, China) was injected into the penile tissue. The injection sites were located approximately 0.5 to 1.0 cm around the penile mass, within the inner or outer foreskin. Three to four injection points were selected. The injection was performed slowly, avoiding blood vessels by aspirating the syringe before the injection. Care was taken to avoid direct injection into the tumor mass.

According to our previous research results [17], we determined that administering carbon nanoparticle injection 30 min before sentinel lymph node biopsy (SLNB) surgery was optimal. For SLNB, the tracer was injected 30 min before surgery. For ILND, the injection occurred 24 to 48 h before surgery to allow the tracer to migrate along the lymphatic vessels. All centres followed the standardized protocol for tracer injection.

#### Lymph node sorting method

In the NLFLNSG, the entire excised tissue specimen was marked by the lead surgeon or assistant to indicate the anatomical regions of the lymph nodes (such as shallow group or deep group). The tissue was carefully dissected, and the lymph nodes were separated from the surrounding fat, soft tissue, blood vessels, and nerves. The dissected lymph nodes were placed in one specimen bag, while the remaining tissue was placed in another. Both specimen groups were sent to the pathology department for analysis.

In the TLNSG, the entire tissue specimen was marked with the anatomical location of the lymph node and sent directly to the pathology department for processing according to the traditional fixation, sorting, and detection procedures. All medical centres adhered to standardized ILND procedures for penile cancer.

### Primary penile cancer cell line establishment, culturing, and passaging

Fresh penile cancer tissue specimens were stored in a sterile preservation solution and processed within 24 h. The tissue was washed, cut into 1 mm³ pieces, and transferred to a centrifuge tube containing serum-free wash solution. The pieces were centrifuged, and serum-free culture medium(Gibco, USA) and digestive enzymes(Hefei PreceDo Medical Laboratory Co., Ltd., Hefei, China) were added for digestion. After filtration and centrifugation, the cell pellet was resuspended in primary cell culture medium (Hefei PreceDo Medical Laboratory Co., Ltd., Hefei, China) and cultured at 37 °C with 5% CO_2_. When the cells reached 80%–90% confluence, they were passaged using trypsin.

### Nude mouse penile cancer lymphatic metastasis animal model

Twelve male BALB/c-NU nude mice JiangSu GemPharmatech Co., Ltd., JiangSu, China) aged 4 to 5 weeks were selected for the study, with 6 in each group of carbon nanoparticles and methylene blue. Penile cancer cell suspensions were injected into the pads on both hind paws of the mice. Tumor formation was observed after 2 weeks, and metastasis was visible in the popliteal/inguinal lymph nodes after 4 weeks. Then, nanocarbon suspension or methylene blue was injected into the paw pad respectively. 30 min or 24–48 h later, the nude mice were dissected to observe the lymphatic tracing effect. 3 nude mice were selected at different injection times. The experimental protocol was approved by the Animal Ethics Committee of Anhui Medical University (Approval No. LLSC20220472). After the experiment, the mice were executed by cervical dislocation method after anesthesia with chloral hydrate. Details of the experiment can be found in the ARRIVE Guidelines Checklist.

### Statistical methods

Normality was assessed with the Shapiro–Wilk test, and homogeneity of variance was evaluated with Levene’s test. Differences among the three groups were analysed using one-way analysis of variance (ANOVA) or the Kruskal–Wallis H test. Pairwise comparisons were performed using the Bonferroni test, Bonferroni-corrected Mann–Whitney U-test, independent samples t-test, or chi-square test. Statistical significance was set at *p* < 0.017 for the Bonferroni-corrected Mann–Whitney U-test and *p* < 0.05 for all other methods. Categorical variables were presented as counts and percentages. Normally distributed continuous data were presented as mean ± standard deviation (x̅ ± s); non-normally distributed data were presented as median (interquartile range). All statistical analyses were performed using SPSS version 20.0 (IBM, USA).

## Results

### Baseline characteristics of patients

A total of 62 patients diagnosed with penile cancer were included in the study. Among them, 33 patients underwent SLNB, involving 65 inguinal surgical sites. The median age of these patients was 70 years (range: 37–85 years). The pathological type was SCC in all cases. Of these, 28 patients (84.85%) had cT1, 5 patients (15.15%) had cT2; 4 patients (12.12%) had cN0, 11 patients (33.33%) had cN1, 17 patients (51.52%) had cN2, and 1 patient (3.03%) had cN3. All patients met the inclusion criteria and were randomly assigned to different groups: the carbon nanoparticle group (11 cases), methylene blue group (10 cases), and the control group (no tracer injection, 12 cases).

**Figure 1.**
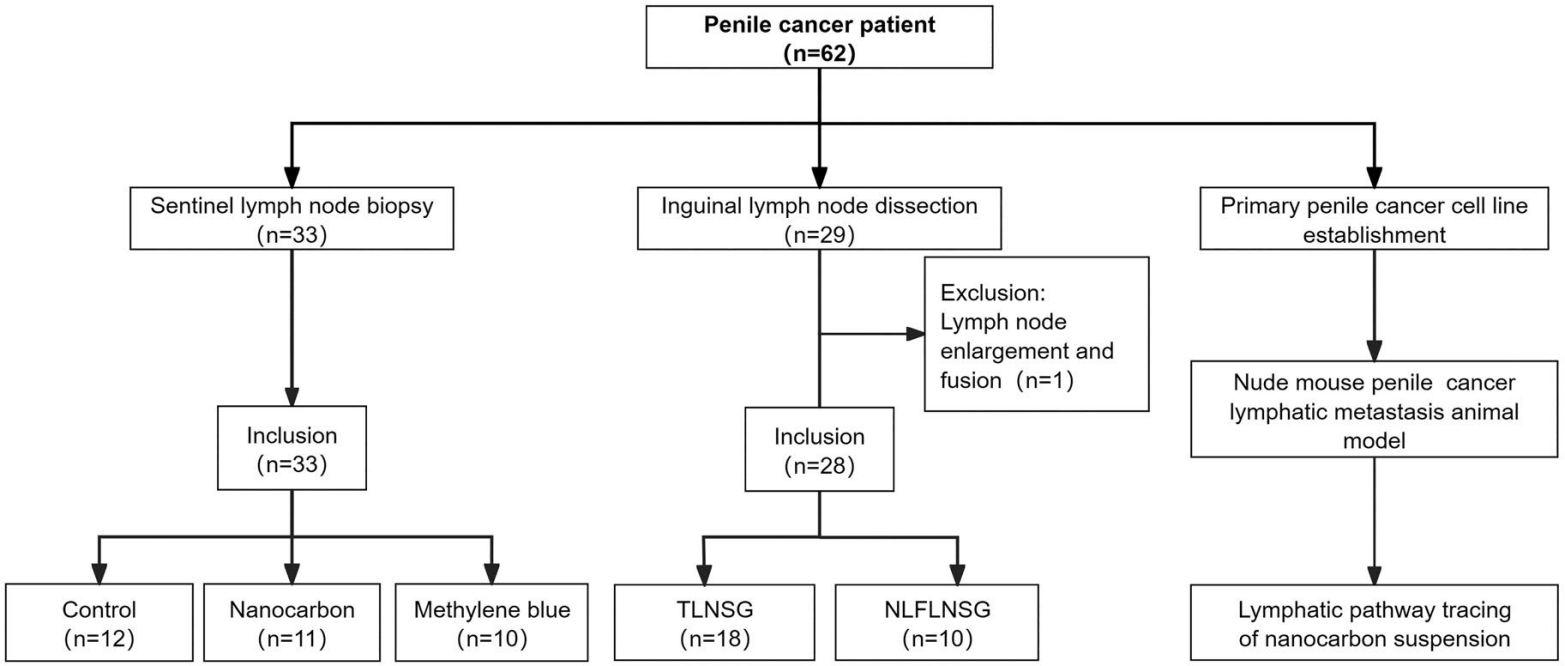
Flowchart of inguinal lymphatic tracing study for penile cancer.

Additionally, 29 patients underwent ILND, involving 44 inguinal surgical sites. The median age of these patients was 66 years (range: 40–87 years). The pathological types included squamous cell carcinoma (26 cases), melanoma (2 cases), and Paget’s disease (1 case). Of these, 22 patients (75.86%) had cT1, 7 patients (24.14%) had cT2; 1 patient (3.45%) had cN0, 7 patients (24.14%) had cN1, 19 patients (65.52%) had cN2, and 2 patients (6.90%) had cN3. All patients were also randomly assigned to different groups. Among the 11 patients in the NLFLNSG, one patient was excluded from the study due to lymph node enlargement and fusion, which made it impossible to accurately count the number of lymph nodes. Therefore, the subsequent data analysis was based on the remaining 10 patients. All 18 patients in the TLNSG met the inclusion criteria. An overview of the research process for the 62 patients is shown in Figure 1, and the summary of baseline characteristics is provided in Table 1.

**Table 1.** Clinical characteristics of 62 penile cancer patients.

Variable	SLNB (*n* = 33)	ILND (*n* = 29)
Number of Inguinal Surgical Sites (NOISS)	65	44
Age at Surgery (yr), Median (IQR)	70 (37–85)	66 (40–87)
**Pathological Type**		
Squamous Cell Carcinoma	33(100.00)	26(89.66)
Melanoma	/	2(6.90)
Paget’s Disease	/	1(3.45)
**cT - Stage**		
T1	28(84.85)	22(75.86)
T2	5(15.15)	7(24.14)
**cN - Stage**		
N0	4(12.12)	1(3.45)
N1	11(33.33)	7(24.14)
N2	17(51.52)	19(65.52)
N3	1(3.03)	2(6.90)
**Group**		
Control Group/TLNSG	12(36.36)	18(62.07)
Nanocarbon Group/NLFLNSG	11(33.33)	11(37.93)
Methylene Blue Group	10(30.30)	/

### Nanocarbon characterization and identification

**Figure 2.**
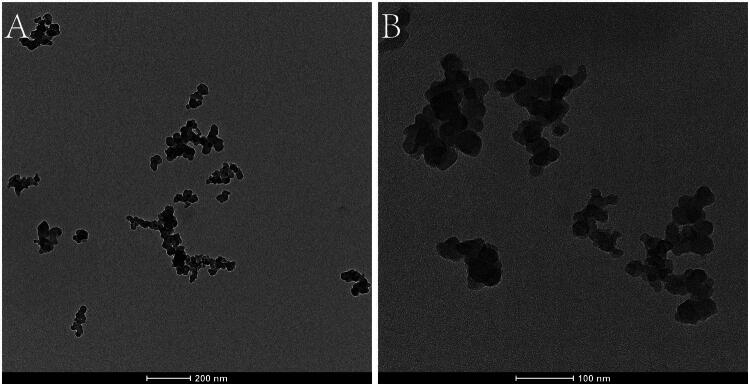
Characterization of nanocarbon using TEM. Individual nanocarbon particles have an approximate size of 20 nm, while the suspension contains aggregated particles with an average size of 150 nm (A, B).

Transmission electron microscopy (TEM) imaging revealed individual nanocarbon particles suspended in the solution each with an approximate size of 20 nm. The suspension also contained aggregated nanocarbon particles, with an average size of 150 nm (Figure 2(A,B)). These aggregates were clearly visible under TEM.

### Nanocarbon suspension and methylene blue are both safe and efficient tracers

**Figure 3.**
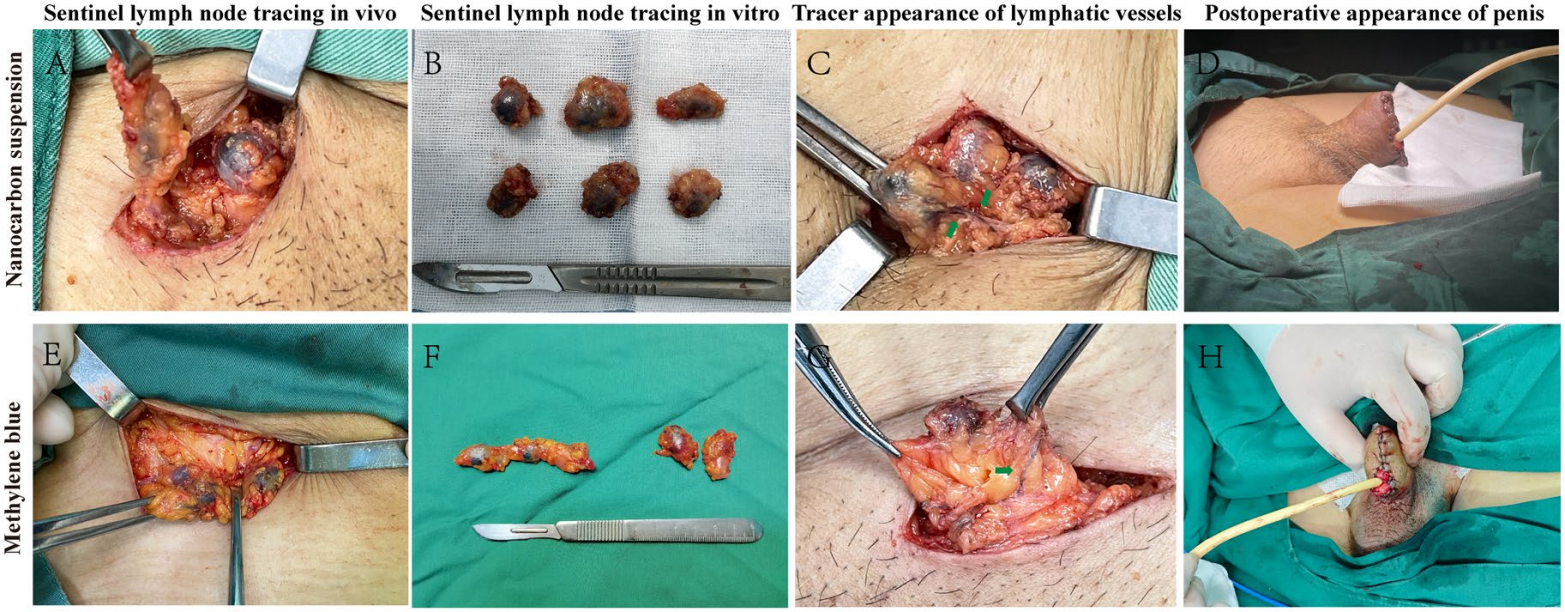
Comparison of the tracing effects between nanocarbon suspension and methylene blue. Both the nanocarbon suspension (A, B) and methylene blue (E, F) can effectively stain the inguinal sentinel lymph nodes of penile cancer in black, dark blue, or light blue. The nanocarbon suspension (C) and methylene blue (G) also demonstrate good effects in lymphatic vessel staining, clearly marking the linear pathways of lymphatic vessels indicated by green arrows. The stained areas formed after the injection of nanocarbon suspension (D) and methylene blue (H) are typically removed together with the penile resection and without adversely affecting the postoperative appearance.

Both nanocarbon suspension(Figure 3(A,B)) and methylene blue(Figure 3(E,F)) effectively function as sentinel lymph node tracers in penile cancer. Nanocarbon suspension and methylene blue stain the lymph nodes in black, deep blue or light blue hues, allowing for easy identification by the naked eye without the need for additional equipment. In addition, the staining effect of methylene blue (Figure 3(G)) in lymphatic vessels is similar to that of nanocarbon suspension (Figure 3(C)), both of which can clearly mark the linear pathways of lymphatic vessels and the outlines of lymph nodes, effectively assist in the rapid identification and removal of sentinel lymph nodes during the operation, and block the lymphatic ducts to minimize the risk of postoperative lymphatic leakage.

The stained areas caused by the injection of nanocarbon suspension (Figure 3(D)) and methylene blue (Figure 3(H)) is generally excised with the penis without additionally affecting the postoperative appearance. Importantly, no adverse reactions or complications were observed in patients treated with either tracer, further supporting their safety and clinical feasibility.

### Methylene blue helps improve surgical efficiency in penile cancer

Our previous study has demonstrated that, compared with the control group, nanocarbon suspension showed significant tracing effects and enhanced surgical efficiency in sentinel lymph node staining of inguinal regions in penile cancer [17]. In this study, by comparing methylene blue with nanocarbon suspension, we evaluated the differences between the two tracers in terms of lymph node detection rate (LNDR), number of lymph nodes per inguinal side (NOLNPIS), positive rate of lymphatic metastasis (PROLM), and operation time (OT). The normality test and homogeneity of variance test for each group’s data were performed using the Shapiro-Wilk test and Levene’s test, respectively. The results showed that the NOLNPIS in the nanocarbon group, methylene blue group, and control group did not follow a normal distribution (P-values of 0.016, 0.006, and <0.0001, respectively), and the assumption of homogeneity of variance was not valid (*p* = 0.001). Further Kruskal-Wallis H test indicated significant differences between the three groups (*p* < 0.0001), and post hoc pairwise comparisons were performed using the Bonferroni-corrected Mann-Whitney U test. Furthermore, the OT in the nanocarbon group, methylene blue group, and control group followed a normal distribution (P-values of 0.110, 0.131, and 0.077, respectively), and the assumption of homogeneity of variance was satisfied (*p* = 0.134). One-way ANOVA indicated significant differences between the three groups (*p* < 0.0001), and post hoc pairwise comparisons were performed using the Bonferroni test.

**Figure 4.**
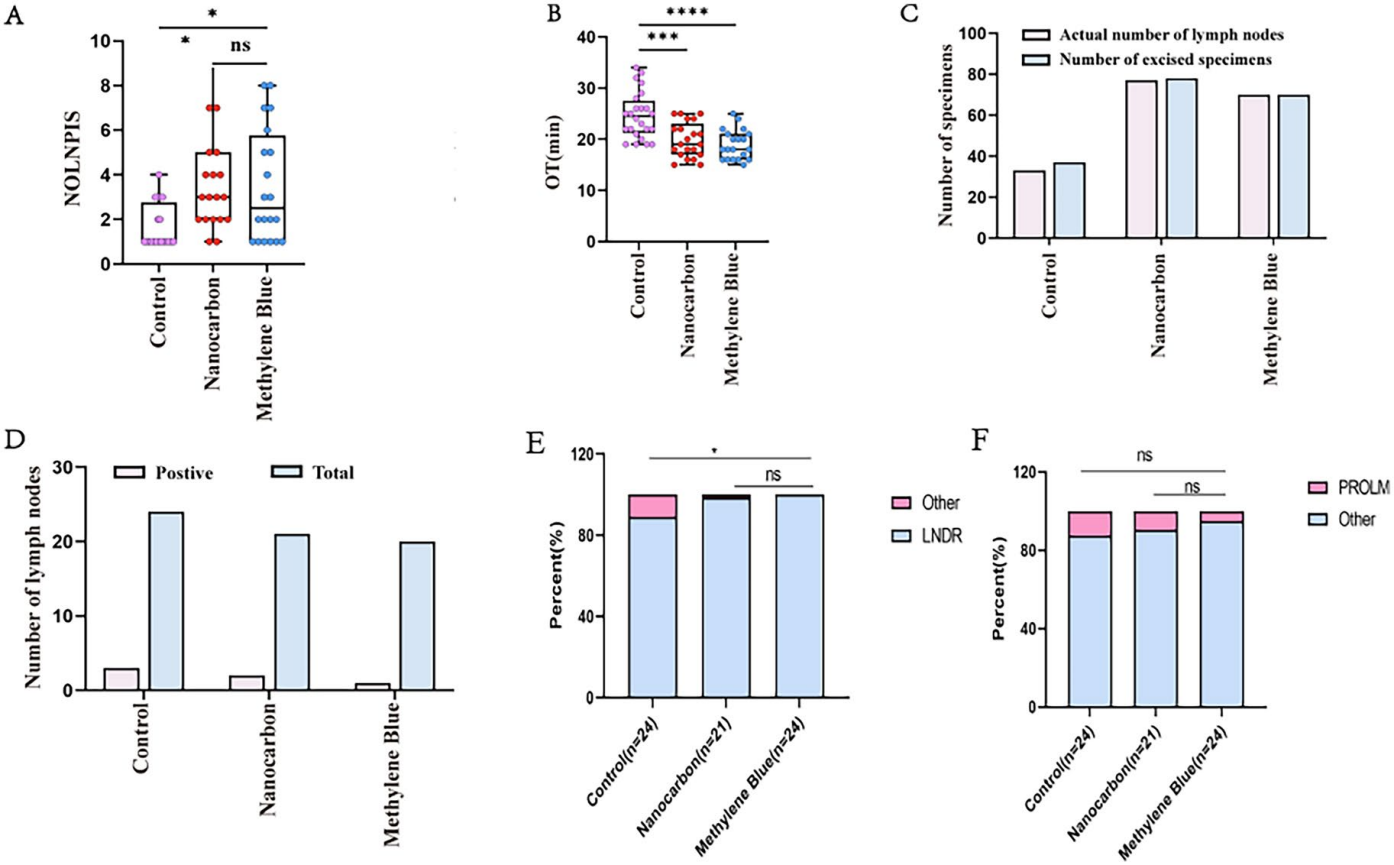
Methylene blue enhances improve the surgical efficiency of sentinel lymph node biopsy for penile cancer. Comparison of the NOLNPIS (A), OT (B), LNDR (C, E), and PROLM (D, F) among the control, nanocarbon, and methylene blue groups. *p < 0.05, **p < 0.01, ***p < 0.001, ****p < 0.0001, ns, not significant.

In the control group, 12 patients underwent surgery in 24 inguinal regions, with 37 lymph node specimens sent for pathological examination, yielding 33 lymph nodes. The LNDR was 89.189% (33/37), with 4 inguinal surgical sites where the sent tissue was confirmed as fibrofatty tissue. The average NOLNPIS was 1.650 ± 0.460, with a PROLM of 12.500% (3/24), and the average OT was 24.667 ± 1.924 min (Table 2, Figure 4).

**Table 2.** Data on various indicators and statistical analysis results for the control group, nanocarbon group, and methylene blue group.

Item	Group		*P* - value	Group		*P* - value
	Nanocarbon Group	Methylene Blue Group		Control Group	Methylene Blue Group	
**NOISS**	21	20	/	24	20	/
**LNDR**	98.718% (77/78)	100.000% (70/70)	*P* = 0.342 χ^2^ = 0.904	89.189% (33/37)	100.0%(70/70)	*P* = 0.013 χ^2^ = 7.860
**NOLNPIS**	3.900 ± 1.134	3.500 ± 1.176	*P* = 0.429 U = 170.500	1.650 ± 0.460	3.500 ± 1.176	*P* = 0.015 U = 111.500
**PROLM**	9.523%(2/21)	5.000%(1/20)	*P* = 0.578 χ^2^ = 0.309	12.500%(3/24)	5.000%(1/20)	*P* = 0.614 χ^2^ = 0.742
**OT (min)**	19.857 ± 1.544	18.950 ± 1.340	*P* = 1.000	24.667 ± 1.924	18.950 ± 1.340	*P* < 0.0001

In the nanocarbon group, 11 patients underwent surgery in 21 inguinal regions, with 78 lymph node specimens sent for pathological examination, yielding 77 lymph nodes. The LNDR was 98.718% (77/78), with 1 inguinal surgical site where the sent tissue was confirmed as fibrofatty tissue. The average NOLNPIS was 3.900 ± 1.134, with a PROLM of 9.523% (2/21), and the average OT was 19.857 ± 1.544 min (Table 2, Figure 4).

In the methylene blue group, 10 patients underwent surgery in 20 inguinal regions, with 70 lymph node specimens sent for pathological examination, yielding 70 lymph nodes. The LNDR was 100.0% (70/70). The average NOLNPIS was 3.500 ± 1.176, with a PROLM of 5.000% (1/20), and the average OT was 18.950 ± 1.340 min (Table 2, Figure 4).

Statistical analysis revealed that, compared with the control group, the methylene blue group showed a significantly higher LNDR (*p* = 0.013, χ^2^ = 7.860), a greater NOLNPIS (*p* = 0.015, < 0.017), and a shorter OT (*p* < 0.0001), significantly improving surgical efficiency, while no significant difference was observed in the PROLM (*p* = 0.614, χ^2^ = 0.742) (Table 2, Figure 4). In comparison with the nanocarbon group, no significant differences were observed between the methylene blue group in terms of LNDR, NOLNPIS, PROLM, or OT (Table 2, Figure 4).

### Construction of primary penile cancer cells

**Figure 5.**
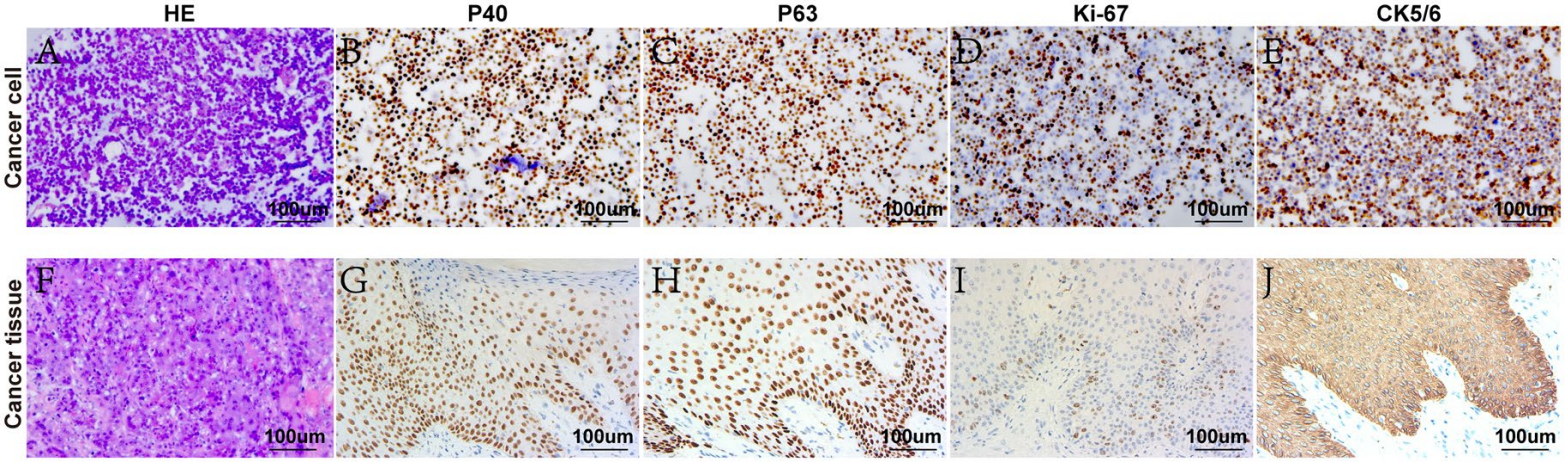
Construction of primary penile SCC cells. HE staining shows that the primary penile cancer cells (A) and tissues (F) exhibit significant atypia. Immunohistochemical analysis indicates the positive expression of SCC markers P40 (B), P63 (C), Ki-67 (D), and CK5/6 (E) in primary penile cancer cells, and P40 (G), P63 (H), Ki-67 (I), and CK5/6 (J) in penile cancer tissues (magnification: 200×).

Primary penile cancer cells were successfully established and cultured from freshly obtained tissue. Hematoxylin and eosin (HE) staining (Figure 5(A,F)) revealed cellular atypia consistent with SCC. Immunohistochemical analysis confirmed the expression of SCC markers, including P40 (Figure 5(B,G)), P63 (Figure 5(C,H)), Ki-67 (Figure 5(D,I)), and CK5/6 (Figure 5(E,J)) in both the primary cells and the tissue. These findings confirm the successful establishment of primary penile SCC cells.

### Nanocarbon suspension exhibits prolonged lymphatic tracing capability

**Figure 6.**
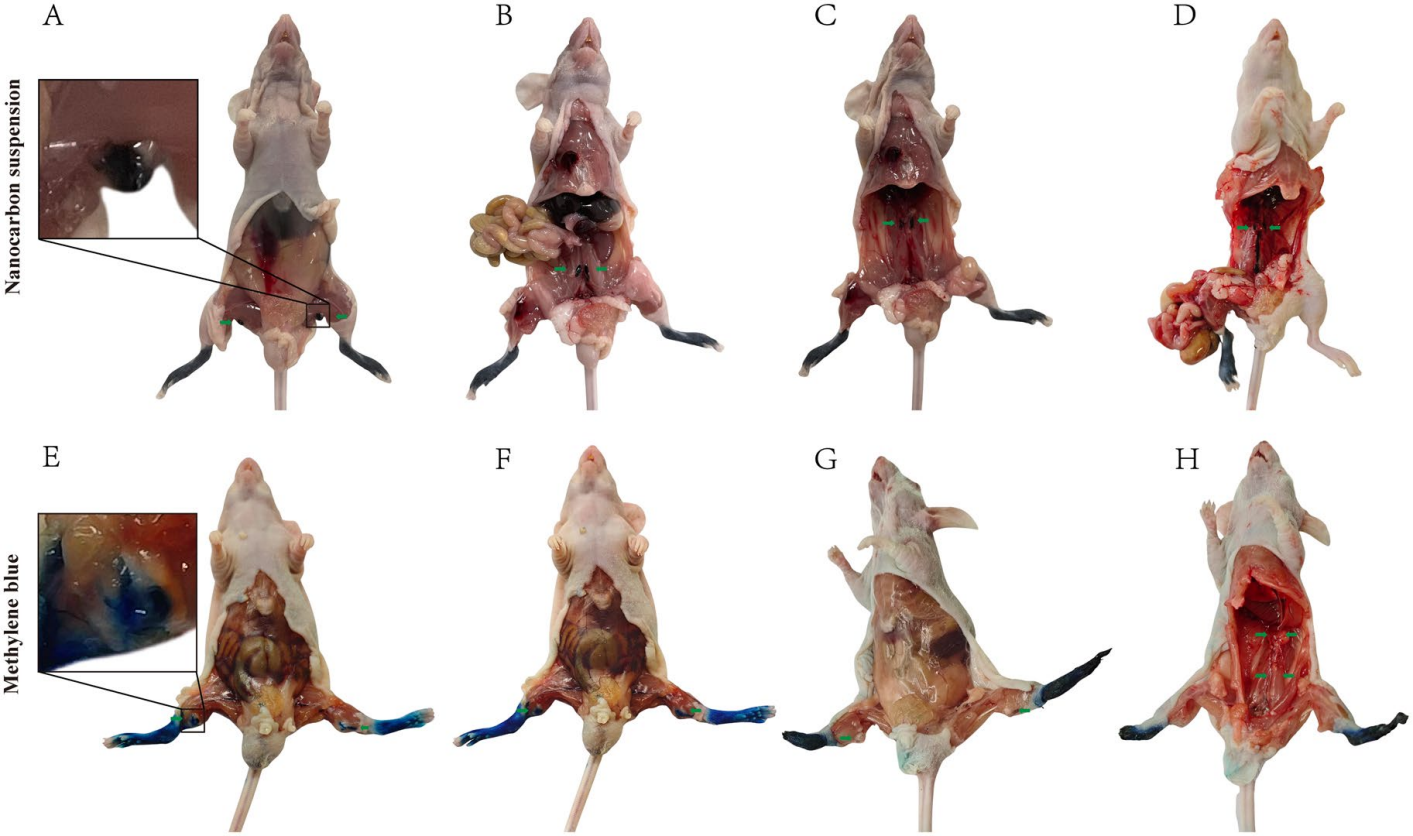
Nanocarbon Suspension Exhibits Prolonged Lymphatic Tracing Capability. Nanocarbon achieves sustained staining in the popliteal/inguinal primary lymph nodes (A), common iliac secondary lymph nodes (B), para-aortic and renal hilum tertiary lymph nodes (C), and intestinal trunk/lumbar trunk/cisterna chyli (D). Following local injection of methylene blue, the dye quickly enters the popliteal/inguinal primary lymph nodes (E, F). However, it fails to achieve sustained staining in the popliteal/inguinal primary lymph nodes (G), common iliac secondary lymph nodes, or para-aortic and renal hilum tertiary lymph nodes (H). Green arrows indicate lymph nodes or lymph node regions.

In a nude mouse model of penile cancer lymphatic metastasis, approximately 30 min after the injection of nanocarbon suspension, nanocarbon was observed in the popliteal/inguinal primary lymph nodes (Figure 6(A)). After 24–48 h, nanocarbon not only remained in the popliteal/inguinal primary lymph nodes but also sequentially entered the common iliac secondary lymph nodes (Figure 6B), the para-aortic renal hilum tertiary lymph nodes (Figure 6(C)), and the intestinal trunk/lumbar trunk/cisterna chyli (Figure 6(D)). The stained lymph nodes exhibited well-defined and intact contours, demonstrating that the nanocarbon suspension possesses stepwise and persistent lymphatic tracing capabilities.

In contrast, approximately 30 min after methylene blue injection, methylene blue was observed in the popliteal/inguinal primary lymph nodes (Figure 6(E)). However, the stained lymph nodes exhibited a ‘spiky sign,’ which may be due to the extravasation and diffusion of methylene blue into the surrounding tissues. This ‘smudging’ phenomenon became more pronounced after approximately 30 min (Figure 6(F)). After 24–48 h, no stained lymph nodes were detected in the popliteal/inguinal primary lymph nodes (Figure 6(G)), common iliac secondary lymph nodes, or para-aortic renal hilum tertiary lymph nodes (Figure 6(H)).

After statistical analysis by chi-square test, when comparing the nanocarbon group and the methylene blue group, there was no significant difference in the staining of the popliteal/inguinal primary lymph nodes of nude mice approximately 30 min after the injection of the tracer (*p* > 0.05). However, the phenomenon of methylene blue permeating and diffusing outside the lymph nodes could be observed. Approximately 24–48 h after the injection of the tracer, nanocarbon staining could still be observed in lymph nodes at all levels, while methylene blue staining could not be observed. There was a statistically significant difference between the two groups in terms of lymph node staining (*p* < 0.0001).

### Nanocarbon suspension tracing combined with fine lymph node sorting increases NOLNPIS

**Figure 7.**
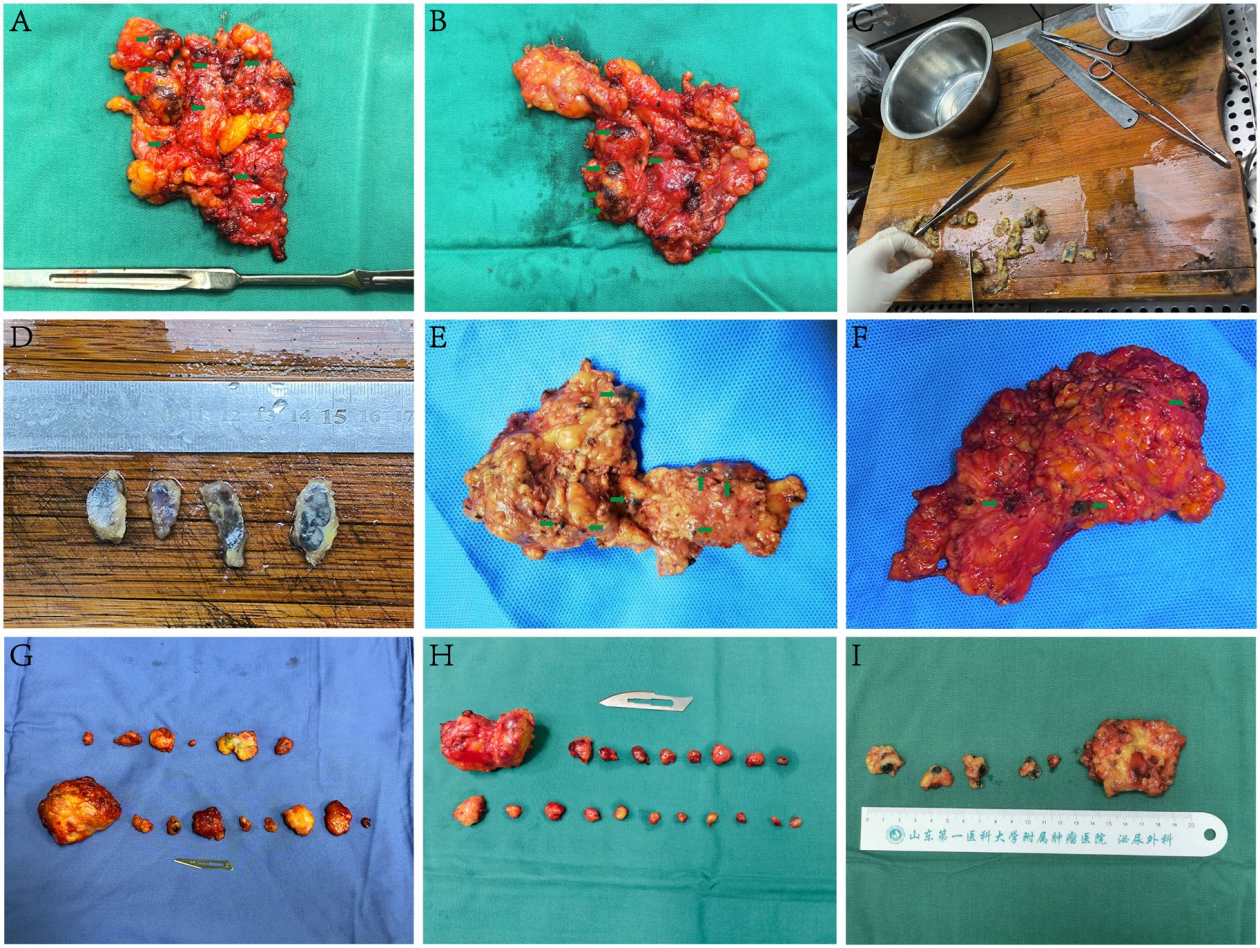
Clinical Application of Nanocarbon Suspension-Assisted Inguinal Lymphadenectomy and Fine Lymph Node Sorting in Penile Cancer. Multiple black-stained lymph nodes are visible on both the anterior and posterior surfaces of the postoperative specimen (A, B). Lymph nodes retrieved by the pathologist from the specimen for analysis (C, D). In human tissue, both superficial and deep lymph nodes are successfully stained black (E, F). During fine lymph node sorting, lymph nodes are individually separated from large adipose tissue blocks (G, H, I). Green arrows indicate black-stained lymph nodes.

In clinical practice, the number of lymph nodes reported in pathological examinations is often lower than expected. To address this issue, we applied a nanocarbon suspension in a patient undergoing ILND for penile cancer. Without thoroughly separating the tissue specimen, an initial estimation based on surface observation from both the front and back of the specimen revealed approximately 10 lymph nodes (Figure 7(A,B)). However, the pathologist identified only 5 lymph nodes (Figure 7(C,D)). This suggests that nanocarbon labelling alone may not be sufficient for accurate pathological staging.

To optimize detection, we combined nanocarbon labelling with fine sorting of pathological specimens. During ILND assisted by nanocarbon lymphatic tracing, after the excision of adipose tissue containing lymph nodes, the surgeon immediately marked the anatomical regions of the lymph nodes. Both superficial and deep lymph nodes were stained black (Figure 7(E,F)), consistent with the findings from animal experiments. Fine lymph node sorting was performed to individually separate the lymph nodes one by one (Figure 7(G,H,I)). We then compared data from the TLNSG and the NLFLNSG. The results showed that the data from the TLNSG deviated from a normal distribution (*p* = 0.011), while the NLFLNSG followed a normal distribution (*p* = 0.586). The assumption of homogeneity of variances was not satisfied (*p* = 0.019), and further statistical analysis used the Mann-Whitney U test. In terms of laparoscopic surgery time, both groups followed a normal distribution (*p* = 0.836 and *p* = 0.792), and the assumption of homogeneity of variances was met (*p* = 0.626), with subsequent statistical analysis using the independent t-test. For open surgery time, both groups also followed a normal distribution (*p* = 0.649 and *p* = 0.530), and homogeneity of variances was satisfied (*p* = 0.204), with further statistical analysis using the independent t-test.

**Figure 8.**
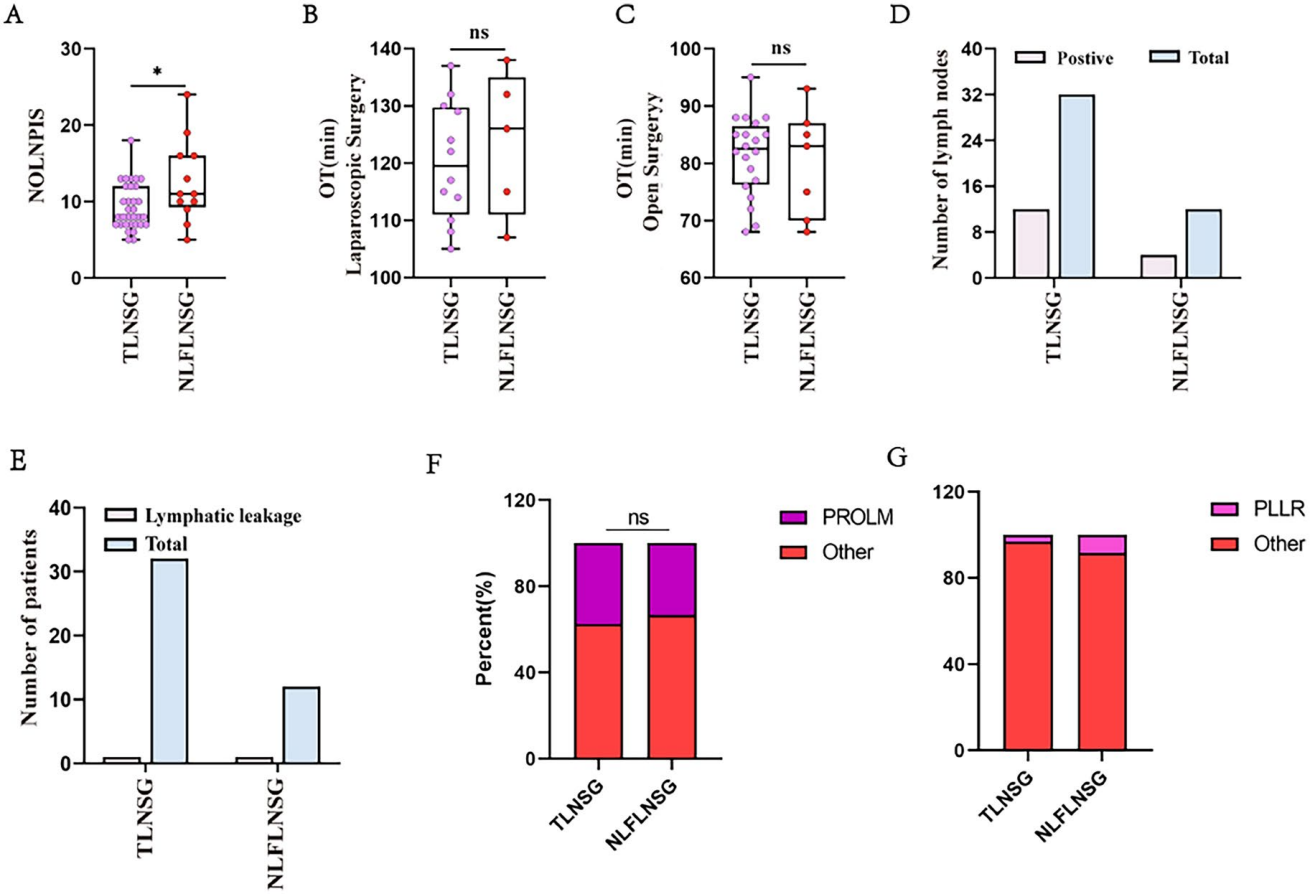
Nanocarbon tracing combined with fine sorting significantly increases the number of detected lymph nodes. Comparison of NOLNPIS (A), laparoscopic operation time (B), open operation time (C), PROLM (D, F), and PLLR (E, G) between the TLNSG and the NLFLNSG. *p < 0.05, **p < 0.01, ***p < 0.001, ****p < 0.0001, ns, not significant.

In the TLNSG, 18 patients underwent 32 inguinal surgical sites, with an average of 9.250 ± 1.059 lymph nodes per site, a PROLM of 37.500% (12/32), laparoscopic surgery time of 120.250 ± 6.559 min, open surgery time of 81.400 ± 3.265 min, and PLLR of 3.125% (1/32) (Table 3, Figure 8).

**Table 3.** Data and statistical analysis results of various indicators for the traditional and fine lymphatic sorting groups.

Item		Group		*P* - value
		TLNSG	NLFLNSG	
**NOISS**		32	12	/
**NOLNPIS**		9.250 ± 1.059	12.583 ± 3.412	P = 0.043, U = 115.000
**PROLM**		37.500% (12/32)	33.333% (4/12)	P = 0.800, χ² = 0.065
**OT**	**Laparoscopic Surgery**	120.250 ± 6.559	123.600 ± 15.590	P = 0.575
	**Open Surgery**	81.400 ± 3.265	80.143 ± 8.604	P = 0.710
**PLLR**		3.125%(1/32)	8.333%(1/12)	P = 0.465, χ^2^=0.546

In the NLFLNSG, 10 patients (after 1 drop-out) underwent 12 inguinal surgical sites, with an average of 12.583 ± 3.412 lymph nodes per site, a PROLM of 33.333% (4/12), laparoscopic surgery time of 123.60 ± 15.590 min, open surgery time of 80.143 ± 8.604 min, and PLLR of 8.333% (1/12). Statistical analysis showed that the NLFLNSG significantly increased the NOLNPIS (*U* = 115.000, *p* = 0.043), but there were no significant differences in PROLM, OT, or PLLR between the two groups (*p* > 0.05) (Table 3, Figure 8).

## Discussion

Lymphatic metastasis is the primary route of spread in penile cancer. The extent of metastasis, the quality of lymph node dissection, and precise pathological staging are all key factors influencing patient survival prognosis. Sentinel lymph node identification and biopsy, as the first site of inguinal lymphatic metastasis in penile cancer, are crucial for accurate staging. Clinically, tracers are commonly used to assist in identifying sentinel lymph nodes. However, agents such as indocyanine green and radioactive 99Tcm-labelled colloid have limitations, including complex procedures, radiation risks, and dependence on costly imaging and detection equipment. Therefore, there is an urgent need to identify a novel, convenient, safe, and effective tracer.

Nanocarbon suspension has become widely used as a tracer in clinical surgery for various tumours. It exists as aggregates with an average particle size of 150 nm. Given that the gaps between endothelial cells of capillaries range from 20–50 nm, and those of capillary lymphatic vessels are range from 120 to 500 nm, nanocarbon suspension is highly lymphotropic and can enter only the lymphatic system, not the bloodstream. This results in black staining of the lymphatic pathways. Previous research has confirmed that nanocarbon suspension can effectively trace inguinal sentinel lymph nodes in penile cancer without the need for additional tracers or devices [17]. However, the high cost of nanocarbon suspension limits its widespread application. Therefore, in this study, we compared methylene blue—a less expensive tracer—with nanocarbon suspension to explore its potential for tracing inguinal sentinel lymph nodes in penile cancer.

Our results showed that both methylene blue and nanocarbon suspension effectively stained the inguinal sentinel lymph nodes and lymphatic vessels, with no significant differences in LNDR, NOLNPIS, PROLM, or OT between the two tracers. However, compared to the control group, the methylene blue group demonstrated a higher detection rate of lymph nodes, retrieved more lymph nodes per inguinal side, and had shorter surgical times. This suggests that methylene blue not only efficiently traces dynamic sentinel lymph nodes but also significantly improves surgical efficiency. The increased detection rate is likely due to the tracer’s ability to assist surgeons in accurately identifying the lymph node contours and locating the linear pathways of lymphatic vessels, thereby reducing the risk of misidentifying fat tissue as lymph nodes. Furthermore, lymphatic tracing facilitates quicker removal of lymph nodes and blockage of lymphatic vessels, potentially reducing postoperative lymphatic leakage.

Importantly, neither of the tracers caused adverse reactions or surgical complications, indicating that both tracers are safe and effective for tracing. Given its lower cost and easier procurement, methylene blue is particularly suitable for use in grassroots hospitals or low-income countries or regions with a high incidence of penile cancer.

For patients undergoing inguinal lymphadenectomy for penile cancer, the number of lymph nodes is crucial for accurate pathological staging and prognosis prediction. An insufficient number of retrieved lymph nodes may lead to underestimation of the pN stage, introducing bias into treatment decisions and follow-up protocols, which could negatively affect prognosis. However, in China and many other countries, postoperative specimens from cancer surgeries are often sorted by pathologists without standardized sampling protocols, leading to missed lymph nodes [18,19]. In Japan, after radical gastrectomy, experienced surgeons immediately perform fine lymph node sorting of specimens, with the average number of lymph nodes obtained reaching 39.4, which makes the number of lymph nodes of gastric cancer surgery in Japan has been in the world’s leading position [20]. Similarly, a study at Memorial Sloan Kettering Cancer Center found that fine lymph node sorting by surgeons significantly increased the number of lymph nodes detected (30 vs. 21, *p* < 0.001) [20]. These data suggest that fine sorting of isolated specimens by experienced surgeons can significantly increase the number of detected lymph nodes, thereby providing more accurate pathological staging and prognosis assessment for patients.

Given that most clinical practices are limited by time and resources, surgeons and pathologists are often unable to simultaneously perform fine lymph node sorting. This makes it difficult for surgeons to distinguish lymph nodes in fatty tissue alone. To address this challenge, we decided to introduce tracers during ILND for penile cancer. This approach enables surgeons to more easily separate the lymph nodes from fat tissue, and subsequently send them to the pathology department for a second examination, reducing the risk of missed lymph nodes during specimen sorting and thereby improving the accuracy of pathological diagnosis. Through this model of lymphatic tracing combined with fine sorting and the sequential sorting by surgeons and pathologists, we aim to further optimize the sorting of pathological specimens and ensure that patients receive the best treatment plan.

While both methylene blue and nanocarbon suspension provided similar visual results in tracing inguinal sentinel lymph nodes, we found that methylene blue does not provide long-lasting staining in animal model experiments. The weak persistence of methylene blue staining means that it can only be applied to lymphatic pathways close to the tumour, such as sentinel lymph nodes. This is likely because methylene blue is a small molecule that easily passes through biological membranes [21–24], diffuses into surrounding tissues, and is absorbed and metabolized. Therefore, in the animal model, the stained lymph nodes exhibited a “spiky sign”. In contrast, nanocarbon suspension persisted in staining sentinel lymph nodes and deeper lymphatic pathways, likely due to its aggregate form (150 nm), which allows for better retention in the lymphatic system. Additionally, nanocarbon’s lymphatic affinity enables it to migrate deeper into the lymphatic system. Our animal experiments and ILND in patients with penile cancer have supported these findings. Therefore, in order to have sufficient time for the tracer to enter all levels of lymph nodes, we choose carbon nanosuspension as the tracer during ILND.

With the assistance of nanocarbon suspension, surgeons followed a fine lymph node sorting protocol. The results showed that the NLFLNSG detected significantly more lymph nodes than the TLNSG (*p* = 0.043). This suggests that combining nanocarbon suspension tracing with fine lymph node sorting improves lymph node detection, enhances the accuracy of pathological staging, and aids in more precise treatment planning, ultimately benefiting patients. Therefore, nanocarbon suspension tracing combined with fine lymph node sorting offers a promising approach to lymph node pathological sorting.

However, no significant differences were observed between the NLFLNSG and the TLNSG in terms of PROLM, OT, or PLLR. The lack of differences in surgical time may be attributed to the fact that, regardless of tracer use, surgery followed standard dissection protocols. Furthermore, the relatively low incidence of penile cancer and the limited sample size may have influenced the results, preventing the full advantages of nanocarbon suspension from becoming apparent in terms of increased PROLM and reduced PLLR. Additionally, postoperative lymphatic leakage is a rare occurrence, and the current sample size may not provide enough statistical power to detect significant differences. To address these limitations, further multicenter prospective cohort studies with larger sample sizes are needed to improve statistical power and adopt standardized surgical and pathological protocols. This will help more accurately assess the clinical value of fine lymph node sorting and provide more reliable evidence for clinical decision-making.

## Conclusion

Both nanocarbon suspension and methylene blue are safe and effective tracers for sentinel lymph node tracing in the inguinal regions of penile cancer. Nanocarbon suspension offers stronger lymphatic retention and provides more durable staining. Its application in fine lymph node sorting after ILND compensates for the lack of surgeon’s experience in fine sorting. This approach helps improve the accuracy of pathological staging and lays the foundation for more precise diagnosis and treatment plans for patients.

## Supplementary Material

Author Checklist Full.pdf

## Data Availability

The data that support the findings of this study are available from the corresponding author upon reasonable request.
